# York County Hospital: "Truth" on the Inquiry

**Published:** 1913-06-21

**Authors:** 


### YORK COUNTY HOSPITAL.

### "Truth" on the Inquiry.

We take the following from *Truth* of June 11, 1913 :—

"The report of Sir Cooper Perry and Mr. Basil Watson upon their inquiry into the charges made against the York Hospital by the late resident medical officers is an exhaustive document. It deals specifically with the twenty-five allegations made. Fourteen of these are found to have had justification, two were admitted, two had some substance, seven were not justified. Amongst those found to be true were the most serious. It was true that a living patient had been removed to the mortuary, that a child had been ill-treated, that the nursing staff was inadequate, that mischarting occurred in the children's ward, and evidence of laxity and neglect in various directions was forthcoming. The inquiry, therefore, justifies not only the publication of the charges in the columns of The Hospital, but confirms in a striking manner the justness of the report on the York Hospital made by Sir Henry Burdett fifteen months previously, in which reorganisation of the administration was shown to be necessary. Sir Henry Burdett's conclusion had been that there was too little touch from the centre with the duties of sister, nurse, and probationer, and that efficiency would be materially increased by the introduction of a controlling hand. No one who reads the Commissioners' report could fail to admit that this criticism erred, if it erred at all, on the side of moderation. In fact, the lack of touch between the management and the staff is pointed out in the final paragraph of the report, which comments upon the ignorance in which the medical residents were kept as to what was being done in regard to complaints by those in authority. Had facilities been afforded the residents of ready access to the chairman and the house committee, the Commissioners are convinced that, whatever good may come of their investigations, 'might equally well have been attained without the trouble, annoyance, and waste of time which this inquiry has entailed.'

"The first thing that is needed, therefore, if scandals are to be averted in the future, and if the York Hospital is to attain to the standard of efficiency expected of any modern medical institution, is the importation into the governing body of some of that common sense which the average man brings to bear on the everyday administration of his own business affairs. No hospital, any more than any other human enterprise, can be carried on efficiently if those engaged in it are treated by the heads as mere automata and neither consulted about changes that seem desirable, nor informed of them when such are decided upon. If the governors had acted upon this common-sense principle there would, according to the Commissioners, have been no York Hospital scandal. It was only when to all appearances the resident medical officers found that their complaints were disregarded and their recommendations unnoticed that they resigned and gave publicity to the matters which they considered, and rightly considered, to merit the gravest consideration. It now appears that the hospital authorities had given these matters, or some of them, their attention, and had even framed rules to provide against such mishaps as the taking of a living patient to the mortuary happening in the future. But what the authorities apparently did not realise, and have so far failed to realise, is that rules alone cannot secure effective administration. The most perfect rules will prove ineffective unless administered by an able and efficient organiser, and to this part of the problem the York Hospital governors must turn their attention if they really want to set their house in order."

